# TRiC Is a Structural Component of Mammalian Sperm Axonemes

**DOI:** 10.1002/cm.22005

**Published:** 2025-02-10

**Authors:** Alan Brown, Miguel Ricardo Leung, Tzviya Zeev‐Ben‐Mordehai, Rui Zhang

**Affiliations:** ^1^ Department of Biological Chemistry and Molecular Pharmacology Blavatnik Institute, Harvard Medical School Boston Massachusetts USA; ^2^ Hubrecht Institute‐KNAW & University Medical Center Utrecht Utrecht the Netherlands; ^3^ Structural Biochemistry Group, Bijvoet Centre for Biomolecular Research Utrecht University Utrecht the Netherlands; ^4^ Department of Biochemistry and Molecular Biophysics Washington University in St. Louis, School of Medicine St. Louis Missouri USA

**Keywords:** axonemes, chaperonin, cryo‐EM, sperm, TRiC

## Abstract

The TRiC chaperonin is responsible for folding ~5%–10% of the proteome in eukaryotic cells. Our recent cryo‐electron microscopy studies of axonemes from diverse mammalian cell types led to the surprising discovery that a fully assembled TRiC chaperonin is a structural component of mammalian sperm flagella, where it is tethered to the radial spokes of doublet microtubules. In contrast, axoneme‐tethered TRiC is not observed in mammalian epithelial cilia, nor in any of the non‐mammalian sperm flagella studied to date. In this Perspective, we explore several hypotheses for the potential functions of axoneme‐tethered TRiC in mature sperm.

TRiC/CCT (TCP1‐ring complex/chaperonin containing TCP1) is a large eukaryotic chaperonin responsible for the proper folding of 5%–10% of cytosolic proteins (Yam et al. 2008), including the cytoskeletal proteins actin and α‐ and β‐tubulin (Sternlicht et al. 1993). TRiC has a distinctive architecture with two stacked rings each consisting of eight paralogous subunits, CCT1 to CCT8, arranged in a precise order conserved across all eukaryotes (Cong et al. 2010). Each CCT subunit has an apical (A) domain that recruits substrate, an intermediate (I) domain, and an equatorial (E) domain that binds ATP and forms the inter‐ring contacts. ATP binding and hydrolysis trigger a conformational change in TRiC, which transitions from an open state that can recruit substrates to a closed state that encapsulates them within its central chamber. Inside the chamber, protein folding occurs in a highly organized and sequential manner, supported by direct interactions with the TRiC subunits (Gestaut et al. 2022).

We recently used single particle analysis electron cryomicroscopy (SPA cryo‐EM) to show that fully assembled TRiC chaperonins are stably incorporated into bovine sperm axonemes, where they are suspended between radial spokes RS1 and RS2 (Leung et al. 2025) (Figure 1a). To our knowledge, this is the first example of TRiC being specifically anchored to a precise subcellular location. This discovery was independently corroborated by a recent preprint (Meng et al. 2024). In situ electron cryotomography (cryo‐ET) observed similar barrel‐shaped densities in mouse, pig, horse, and human sperm axonemes (Chen et al. 2023; Gadadhar et al. 2021; Leung et al. 2021), and classical EM studies likely detected TRiC even earlier in rat spermatozoa (Olson and Linck 1977), suggesting that TRiC is a common feature of (eutherian) mammalian sperm axonemes. In contrast, TRiC chaperonins are absent from the axonemes of mammalian epithelial cilia (Leung et al. 2025; Lin et al. 2014; Meng et al. 2024; Walton et al. 2023) and have not been detected in zebrafish (Yamaguchi et al. 2018), sea urchin (Lin and Nicastro 2018), or eel (Schrad et al. 2025) sperm flagella.

**FIGURE 1:**
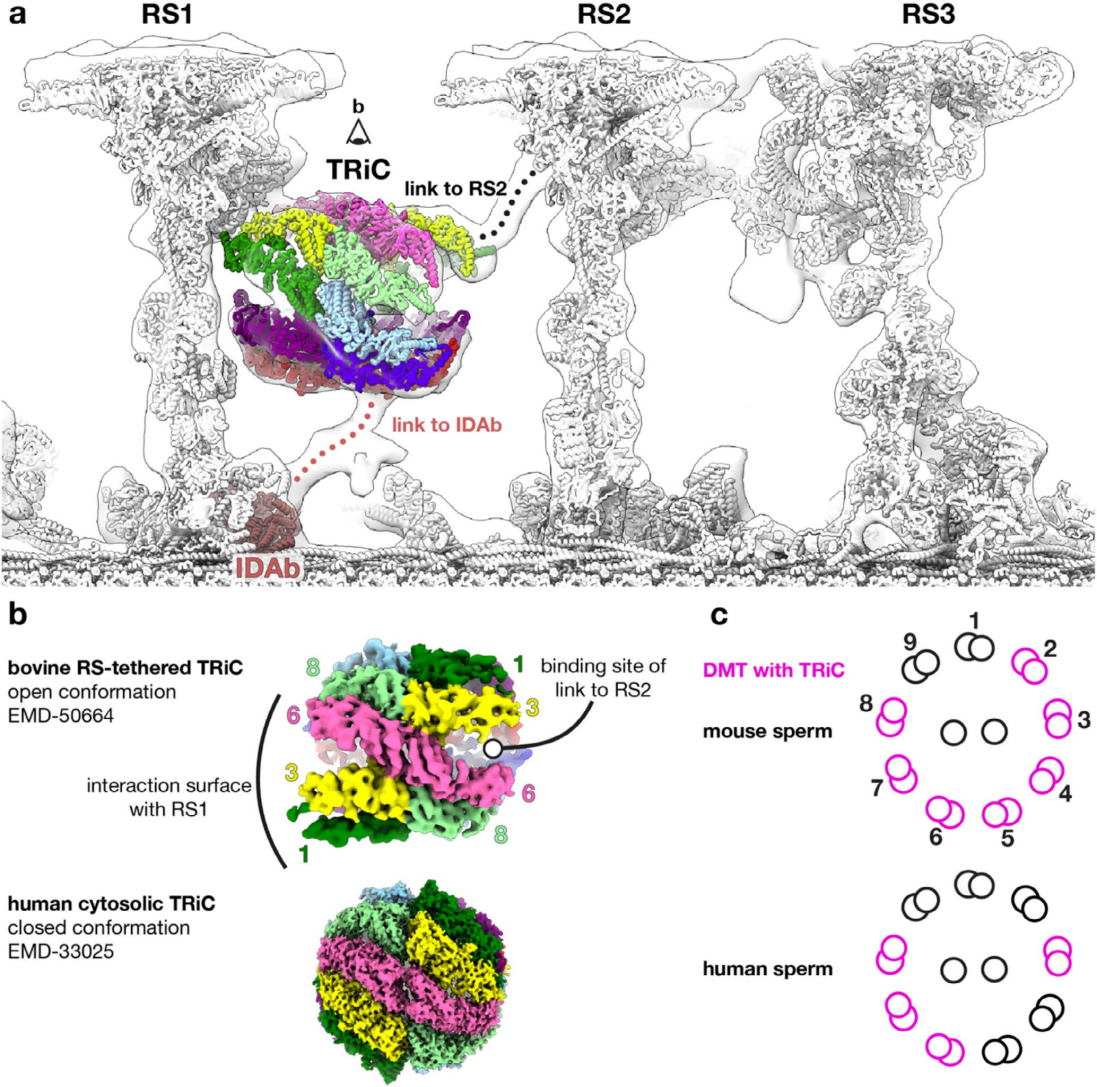
TRiC is tethered to radial spokes in mammalian sperm axonemal doublet microtubules (DMTs). (a) An atomic model of the 96‐nm repeat of bovine sperm DMTs with TRiC colored by individual CCT subunit. The model is fitted into an in situ subtomogram average of DMT7 from mouse sperm axonemes (Chen et al. 2023). Proteins linking TRiC to RS2 and IDA*b* are yet to be identified. (b) Cryo‐EM maps comparing bovine RS‐tethered TRiC in the open state (EMD‐50664) (Leung et al. 2025) with human cytosolic TRiC in the closed state (EMD‐33025) (Liu et al. 2023). (c) Schematic cross sections of mouse and human sperm axonemes, with pink indicating specific DMTs to which TRiC is bound (Chen et al. 2023).

The unique presence of TRiC in mammalian sperm axonemes raises interesting questions as to its function. One hypothesis proposed by Meng et al. (2024) is that axoneme‐tethered TRiC facilitates the folding of proteins synthesized locally within the sperm flagellum. This is a provocative hypothesis because mature spermatozoa as well as flagella/cilia are conventionally thought to be translationally silent. There are indeed data that challenge this dogma, including the observation of many ribosomal proteins and translational factors in the proteomes of mature spermatozoa. However, complete sets of ribosomal proteins are rarely observed, and ribosomal proteins are almost completely absent in mass spectrometry of porcine spermatozoa (Leung et al. 2021). Multiple studies also fail to detect intact ribosomal RNAs in purified spermatozoa, although fragments are present (Johnson et al. 2011; Sellem et al. 2020), and intact ribosomes are yet to be visualized inside sperm flagella or any other type of cilium by cryo‐ET. An alternative scenario is that axoneme‐anchored TRiC only acts as a chaperone during spermiogenesis and serves no functional purpose in mature spermatozoa. Further work on the spatiotemporal distribution of TRiC during spermiogenesis, and on the assembly process of the sperm tail more generally, will help clarify this possibility.

If TRiC does not function to fold newly synthesized proteins in the mature sperm, might it instead repair existing proteins? After ejaculation, mature spermatozoa remain motile anywhere from several hours up to several days for some mammals (Alves et al. 2021). During this period, damage to the axoneme—particularly to the tubulin lattice—may occur, either due to the direct action of dynein motors stepping on microtubules or due to bending stress. In other types of cilia, replacement proteins are synthesized in the cytoplasm and delivered to the site of damage by intraflagellar transport (IFT) (Lechtreck et al. 2018). Since IFT does not operate in mature spermatozoa (San Agustin, Pazour, and Witman 2015), a TRiC‐based repair mechanism has the potential to provide an evolutionary advantage. However, this model relies on two assumptions that have no direct experimental evidence: first, that damage to axonemal microtubules occurs as sperm swim; and second, that TRiC can refold proteins arriving in states fundamentally different from unfolded, newly synthesized proteins.

Both proposed models assume that axoneme‐tethered TRiC is a functional chaperone; a corollary to this is that TRiC would need to transition between open and closed states. Yet, axoneme‐anchored TRiC has only been observed in its open conformation, contrasting with cryo‐ET analysis of cytosolic TRiC in HEK293 cells, where over 50% of TRiC particles exist in the closed state (Xing et al. 2024). The tethers that suspend TRiC between the radial spokes may prevent TRiC from conformationally cycling as they involve interactions with the apical domains of CCT3 and CCT6 **(**Figure 1b **)**. If these interactions fail to survive the transition to the closed state, could TRiC fall off the radial spokes when actively chaperoning a (re)folding protein? This hypothesis predicts that it should be possible to observe detached [closed] TRiC complexes in tomograms of mammalian sperm flagella, which to our knowledge has not been reported.

If tethering to the axoneme renders TRiC incapable of folding or refolding proteins, could it function as a mechanical regulator instead? This would represent a fascinating case of molecular exaptation. In situ cryo‐ET of mouse and human sperm flagella found that TRiC complexes were asymmetrically distributed around the axoneme, though the precise pattern of asymmetry differed between species (Chen et al. 2023) (Figure 1c). This asymmetric distribution raises the possibility that TRiC contributes to the asymmetric, non‐planar waveforms of mammalian spermatozoa. How this could be achieved is open to speculation. One possibility is that the TRiC limits the tilt of radial spokes during flagellar beating (Warner and Satir 1974), subtly influencing the waveform. Subtomogram averages suggest that TRiC interacts indirectly with one of the axonemal dyneins (IDA*b*) (Chen et al. 2023; Meng et al. 2024) (Figure 1a). The proteins mediating these connections remain unidentified, but they may help propagate structural changes from the radial spokes through TRiC to other regulatory elements of the axoneme.

## 1 Future Directions

Defining TRiC's role in the sperm tail will require extensive follow‐up experiments, involving both pharmacological and genetic perturbation. Inhibiting TRiC activity in mature sperm, with and without translational inhibitors, should test whether its (re)folding activity impacts sustained swimming and/or capacitation. Identifying and genetically ablating the proteins that tether TRiC to radial spokes should test whether anchoring TRiC to the axoneme has any impact on the flagellar waveform, although it will be challenging to determine the underlying mechanism. Exogenously expressing these tethering proteins in other cilia, for instance in an airway culture model, could test whether they are sufficient to target TRiC to the axoneme and whether this targeting affects ciliary function. Higher‐resolution structures of axoneme‐tethered TRiC are needed to determine whether the testis‐specific paralogs CCT6B and CCT8L2 are present and contribute to the specific anchoring or orientation of TRiC. Genetic ablation of these paralogs would test their necessity for spermatogenesis, as is the case for testis‐specific CCT subunits in flatworms (Counts, Hester, and Rouhana 2017). Finally, continued structural exploration of native axonemes from other species, cell types, and experimental conditions will address whether TRiC associates with the axoneme in other contexts, perhaps transiently during ciliary growth or regeneration.

## Conflicts of Interest

The authors declare no conflicts of interest.
